# A Honeycomb Film Template-Based Method for High-Throughput Preparation of Anti-*Salmonella typhimurium* 14,028 Phage Microgels

**DOI:** 10.3390/ijms252211911

**Published:** 2024-11-06

**Authors:** Jing Wu, Tingtao An, Yaxiong Song, Shuo Wang

**Affiliations:** 1 School of Medicine, Nankai University, Tianjin 300071, China; wujing2020@nankai.edu.cn (J.W.); ysong90@nankai.edu.cn (Y.S.); 2 College of Food Science and Engineering, Tianjin University of Science and Technology, Tianjin 300071, China; tingtaoan@163.com

**Keywords:** honeycomb film template, phage microgel, high-throughput, *Salmonella typhimurium*, food biological control

## Abstract

Developing efficient anti-microbials for thoroughly addressing *Salmonella* contamination is essential for the improvement of food safety. Phage-built materials have shown great potential for biocontrol in environments. Due to challenges in delivery and stability, their widespread use has remained unattainable. Here, we have developed a honeycomb film template-based method for the high-throughput preparation of phage microgels. The honeycomb film template can be simply fabricated in a humid chamber based on a well-established breath figure method. The bacteriophage microgels can be further manufactured by dropping a pre-gelation solution containing bacteriophages into a honeycomb film template. This method can produce over 210,000 phage microgels in every square centimeter template with each microgel containing 1.04 × 10^7^ phages. They can kill 99.90% of the contaminated *S. typhimurium* 14,028 on chicken samples. This simple, heat-free, and solvent-free method can maintain the strong anti-bacterial efficiency of phages, which can expand the wide application of phage-built microgels for food decontamination.

## 1. Introduction

*Salmonella* is a Gram-negative rod with flagella around it. It is one of the most important pathogens that cause diverse food poisoning symptoms and is commonly associated with the consumption of contaminated foods [1]. Farmers’ intensive practices, centralized food processing, and dense urbanization have enhanced *Salmonella*’s growth and spread. Furthermore, advancements in food production technology, along with the introduction of novel raw materials and equipment, have complicated food contamination issues [2]. Moreover, it is difficult to completely cure *Salmonella* since it contains many drug-resistant strains and antibiotic resistance is increasing. To improve food safety and protect people’s health, developing efficient anti-microbials for thoroughly addressing *Salmonella* contamination is the main solution.

There is a wide range of packaging materials for food preservation, including hydrogel membranes, absorbents, and silver-based materials [3,4]. These materials can delay the deterioration of goods, extend shelf life, and maintain food quality and safety. However, these materials are not suitable for environmental and equipment contaminants due to their relatively low anti-bacterial efficiency and potential harm to the environment. Bacteriophages have recently become widespread biocontrol agents that specifically target and destroy harmful bacteria [5,6]. In environments with existing beneficial bacterial populations, such as food products, agriculture, or human therapies, phages can eliminate harmful bacteria without disrupting the balance of these communities. Using phages for food safety is advantageous because, unlike most anti-microbials, they do not alter the taste, texture, or nutritional quality of food and can safely decontaminate food from farm to consumer. Moreover, phages as biocontrol agents can minimize secondary infections and side effects because they target specific host bacteria until they are destroyed [7]. Various phage-built materials, including bulk gels, fibers, microspheres, and films have been developed for the biocontrol of *Listeria monocytogenens*, *Salmonella*, and *Escherichia coli O157:H7*, indicating promising prospects in food preservation and environmental contaminations [8]. Although phage-built materials are promising, their widespread use remains elusive, partly because of challenges with delivery and stability that limit their effectiveness.

In this study, we created whey protein and trehalose crosslinked phage microgels using a custom, biologics-friendly, high-throughput polystyrene mold. The hydrogel’s natural hydration protects phages from drying out [9,10], while its anti-bacterial properties work with the phages to boost anti-bacterial effects [11,12,13]. Microgels exhibit improved flow properties, making them suitable for aerosol or injection delivery. The template allows for loading concentrated phages into microgels, enhancing their anti-microbial activity and bio-functionality by forming hybrid protein phage microgels. Each microgel contains half a million cross-linked phages organized in aligned bundles. Microgel spray and patches were created to test their anti-microbial effectiveness in preventing bacterial infections in chicken meat. While phage-based hydrogels are known for food preservation, phage-based microgels might offer significant potential for food decontamination.

## 2. Results and Discussion

### 2.1. Scheme of the Preparation of Bacteriophage Microgels

**Figure 1:**
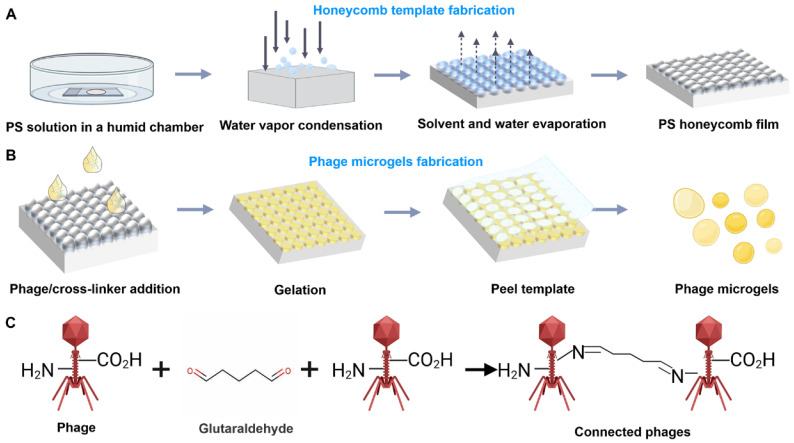
Scheme of the preparation of bacteriophage microgels using honeycomb film as a template. (**A**) A schematic illustration of the process for the preparation of honeycomb templates. (**B**) Schematic image of preparing phage microgels: Honeycomb film is plasma-coated to increase hydrophilicity; Phage and crosslinker mixture solution are cast on the film and placed in the vacuum; The top layer of the film is peeled off using adhesive tape; The phage microgels are isolated after removing the film. (**C**) Crosslinking reactions of bacteriophages with glutaraldehyde (GA).

The bacteriophage microgels were manufactured by dropping a pre-gelation solution that contained bacteriophages into a honeycomb film template. The template is a thin flexible polystyrene film that contains uniform open-ended spherical micropores throughout the film surface (Figure 1A). These films are prepared using the breath figure method, a well-established technique [14,15]. By using this method, large-scale templates with single-layer, closely packed, and homogeneous micropores can be fabricated rapidly and easily. After casting the bacteriophage solution into the honeycomb film, the redundant phage solution was removed from the template surface by a glass slide. Further, the film was transferred into a sealed humid container at 4 °C for 1 day for gelation (Figure 1B). A piece of adhesive tape can be stuck on the composite honeycomb film surface to peel off the top half of the pores. As a result, the microgels inside the film can be exposed to the bottom film layer without damage. Further, the microgels can be isolated from the honeycomb film, followed by dissolution in the water that was used for spray. The gelation of phage aqueous suspension is based on the crosslinking reaction between phage and a chemical crosslinker glutaraldehyde (GA) (Figure 1C). A phage’s protein coat contains abundant amine and carboxyl groups, making it ideal for crosslinking [16,17]. A GA molecule reacts with two amino groups on two phage capsids and forms Schiff bases that connect these two phages [18,19]. Previous reports have demonstrated that adding BSA (bovine serum albumin) to the microgel system can accelerate the gelation process from 12 h to 30 min, leaving insufficient time for phages to self-assemble [12]. Moreover, the abundant amino groups and carboxyl groups offered by BSA can consume excessive crosslinker molecules, minimizing the intramolecular crosslinking within phages and eventually protecting the bioactivity of phages. We, thus, finally added BSA and GA to prepare the phage microgels.

### 2.2. Characterizations of the Prepared Honeycomb Film and Phage Microgels

**Figure 2:**
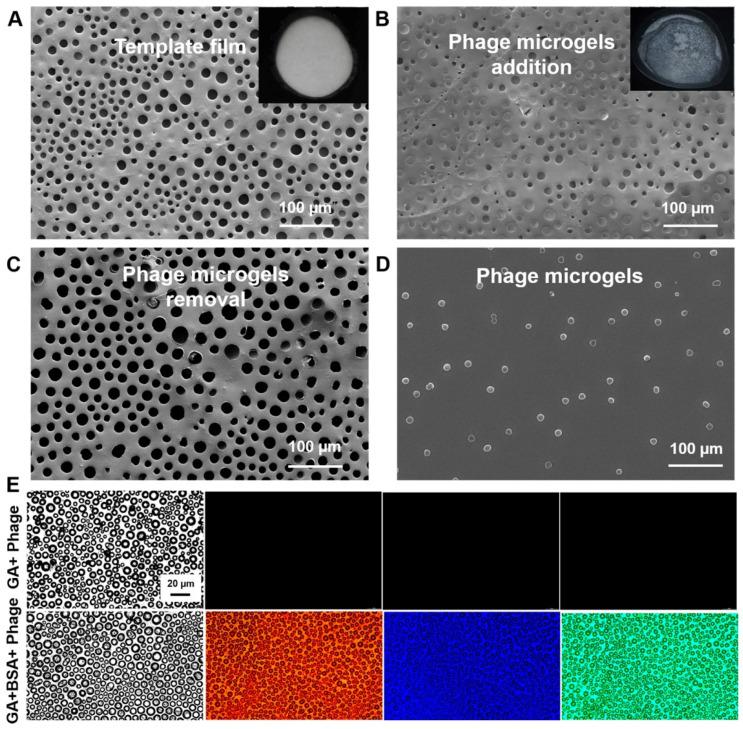
Characterizations of honeycomb film and phage microgels. (**A**–**D**) Surface micromorphologies of the polystyrene (PS) honeycomb film, phage microgel-containing PS film, phage microgel-removed PS film, and isolated phage microgels, which are characterized by surface scanning microscope (SEM) (scale bar 100 mm). The inset photos in (**A**,**B**) are the PS film template and the phage microgel-containing PS film. (**E**) Fluorescent images of honeycomb films after the gelation of phage solutions with GA and GA + BSA. Scale bar: 20 mm. Different fluorescent channels are bright field; red channel (excited at 625 nm); blue channel (excited at 340 nm); and green channel (excited at 465 nm).

To create the honeycomb film template, we prepared a 5% PS (polystyrene) solution (dissolved in chloroform) and distributed it on a pristine glass side within a humidity chamber. The uniform open-ended spherical micropores through the film surface characterized by SEM suggested that the honeycomb film template had been successfully prepared using the breath figure method (Figure 2A). After the treatment of the film with plasma, the phage suspension containing phages and crosslinkers was cast on the film in a vacuum-drying oven with a low-pressure environment for gelation. After gelation for 12 h, the original phage suspension inside the pores successfully turned into an ordered array of solid phage microgels (Figure 2B). After the top half of the pores were peeled off from the honeycomb film with adhesive tape, the gelatinized phage microgels remained inside the pores of the film without damage (Figure 2C). By immersing the film in sterile water with sonication, phage microgels were conveniently isolated from the template (Figure 2D), proving the microgel array inside the template is detachable. In addition, the phage microgels made with GA and BSA showed significant autofluorescence in blue, red, and green channels, due to the electronic transitions such as the n–π* transitions of C=N in the Schiff’s base generated from the crosslinking reaction [20]. The autofluorescence phenomenon potentiates the non-destructive imaging capability of the microgels, allowing for further microgel count and preparation efficiency assessment (Figure 2E).

### 2.3. Preparation Efficiency of Phage Microgels

**Figure 3:**
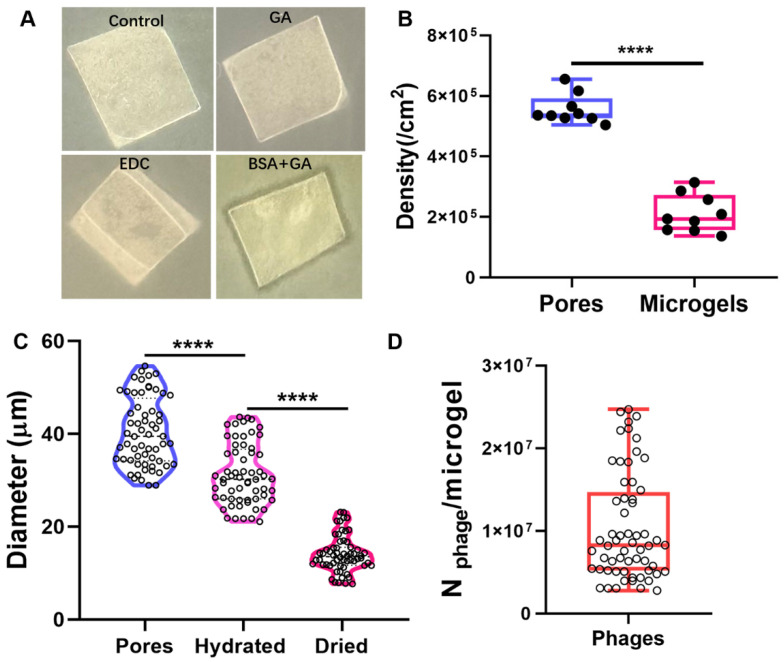
Assessment of the preparation efficiency of phage microgels. (**A**) The plaque inhibition of phage microgels with GA, EDC, and BSA+GA as crosslinkers on the lawn of *S. typhimurium* 14,028. (**B**) Pore density of the template and produced microgel count from every square centimeter of the template (*n* = 9 independent films). (**C**) Size distribution of the template pores, hydrated microgels, and dried microgels (*n* = 20 pores measured 3 independent templates). (**D**) Calculation of the number of phages per microgel using BSA+GA as crosslinker. Box plots show minimum to maximum (whiskers) with all data points. Data are mean ± s.d. Significance was calculated by one-way ANOVA with Tukey multiple comparisons test; **** *p* < 0.0001.

We first compared the anti-microbial activities of phage microgels gelatinized using 1-Ethyl-3-(3-dimethylaminopropyl) carbodiimide (EDC), GA, and BSA + GA as crosslinkers. The undetached microgel arrays in the templates were directly used as an anti-microbial patch to infect *S. typhimurium* 14,028 (Figure 3A). It was found that only the BSA + GA hybrid microgel patch formed an obvious lysis zone around the edges on a lawn of *S. typhimurium* 14,028, showing higher infectivity to host bacteria than others. The isolated phage microgels crosslinked with BSA+GA also formed obvious lysis zones on a lawn of *S. typhimurium* 14,028, confirming their high anti-bacterial efficiency. Further, we investigated the preparation efficiency of phage microgels crosslinked using BSA + GA by calculating the microgel size and density obtained from every square centimeter of the template through ImageJ (Figure 3B,C). The diameter of the hydrate phage microgel was 31.47 ± 6.43 mm, which was comparable with the inner diameter of the template pores (40.35 ± 7.29 mm, unpaired t-test analysis, ns, *p* value = 0.3843). However, after drying in a vacuum, the diameter of the phage microgel decreased to 14.17 ± 3.79 mm. The significant water loss may affect the anti-bacterial activity of phage. We have investigated the storage stability of the dried microgels in further experiments. The density of phage microgels in the film reached 2.10 × 10^5^ ± 5.85 × 10^4^, which was lower than the collected number (2.10 × 10^5^) of isolated phage microgels from a 1 cm^2^ section of PS film (Figure 3B). This could result from the partial filling of phage suspension into the pores or the loss during the isolation process. Based on the phage concentration used (5.7 × 10^14^ PFU/mL) and the average microgel size (31.3 ± 6.51 mm) (Figure 3C), we estimated that each phage microgel is composed of over 1.04 × 10^7^ ± 6.43 × 10^6^ phages (Figure 3D). In summary, we obtained over 2.10 × 10^5^ phage microgels from every square centimeter of our template, where each microgel contains more than 1.04 × 10^7^ phages.

### 2.4. Targeted Anti-Microbial Functions of Phage Microgel Patches and Sprays

**Figure 4:**
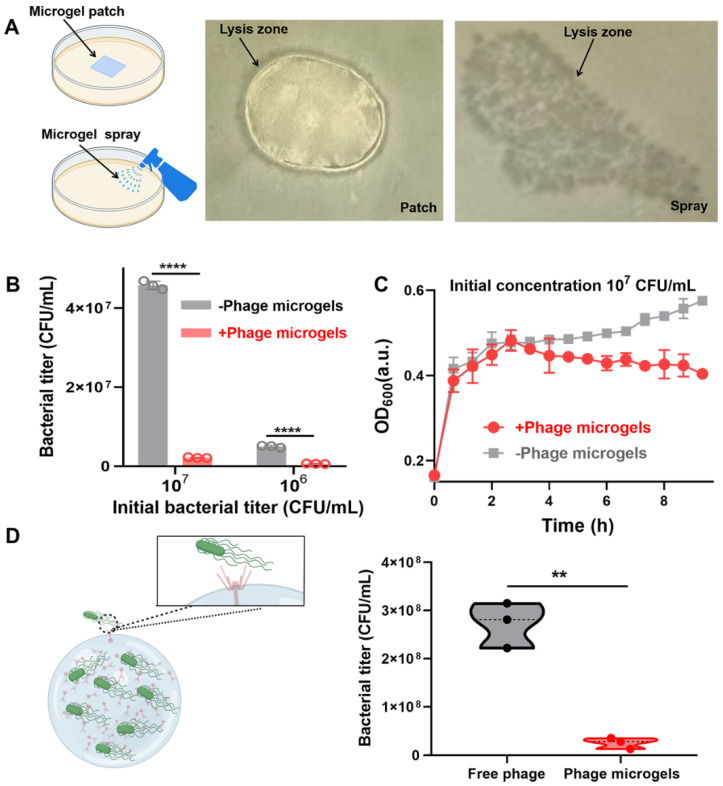
Assessment of anti-microbial activities of phage microgels. (**A**) Photos of phage microgel-embedded patch and phage microgel spray forming lysis zones on the lawn of *S. typhimurium* 14,028. (**B**) Final titer count of *S. typhimurium* 14,028 incubated with and without phage microgels in PBS after 9 h at the initial bacteria concentrations of 10^7^ and 10^6^ CFU/mL. (**C**) Kill curves for *S. typhimurium* 14,028 suspension incubated with and without phage microgels in TSB lawn for 9 h at the initial concentration of 10^7^ CFU/mL. (**D**) Schematic of a phage microgel binding to the host bacteria. (left) Final titer count of *S. typhimurium* 14,028 incubated with phage microgels and free phage in TSB after 9 h at 10^7^ CFU/mL (right) (*n* = 3 independent experiments per bacterial concentration). ** *p* < 0.01, **** *p* < 0.0001.

To confirm the infectivity of the prepared phage microgels, we evaluated the bacteria-killing ability of microgels in solid medium and liquid. An undetached microgel array in the template as an anti-microbial patch and a microgel sprayer were added or sprayed on the surface of *S. typhimurium* 14,028 lawns (Figure 4A). The microgel spray directly used our microgels suspension, containing over 3.10 × 10^4^ microgels per milliliter. Page hybrid microgels maintained their infectivity and the corresponding patch formed a lysis zone around the edges on a lawn and sprayed microgels formed plaques on the bacteria lawn, clearly indicating anti-microbial activity (Figure 4B,C). The nutrient-deficient PBS and nutrient-rich tryptic soy broth (TSB) were used to incubate phage microgels to evaluate their anti-bacterial activity in liquid. Phage microgels were added to the diluted bacterial suspensions at a final concentration of ~310 microgels/mL. *S. typhimurium* 14,028 maintained the same concentration level in PBS without microgels after 9 h. When microgels were added, they killed 95.3 ± 0.28% and 87.8 ± 0.62% of bacteria after 9 h when the initial concentrations of *S. typhimurium* 14,028 in PBS were 10^7^ and 10^6^ CFU/mL, respectively (Figure 4B). In nutrient TSB, phage microgels also showed the ability to prevent bacterial growth. Phage microgels prevented the growth of *S. typhimurium* 14,028 from 3 h, maintaining the bacterial titer at about 10^6^ CFU mL^−1^ while all the controls reached 10^7^ CFU mL^−1^ (Figure 4C, Figure S1 and S2). In summary, phage microgels displayed excellent anti-microbial ability regardless of nutrients and nutrient-deficient environments. We also compared the anti-bacterial activity of phage microgels and free phage suspension. After coculturing with phage microgels for about 150 min, the OD_600_ for *S. typhimurium* 14,028 reached 0.40 ± 0.01, which was lower than that of the phage suspension group (0.47 ± 0.01) (Figure S3). The final concentration of *S. typhimurium* 14,028 incubated with phage microgels (2.56 × 10^7^ ± 9.34 × 10^6^ CFU/mL) for 9 h was also lower than that of the phage suspension group (2.73 × 10^8^ ± 3.84 × 10^7^ CFU/mL) (Figure 4D). The high infectivity of phage microgels may be because the abundant amino groups and carboxyl groups offered by BSA consumed excessive crosslinker molecules, minimizing the intramolecular crosslinking within phages and eventually protecting the bioactivity of phages.

### 2.5. Specificity and Storage Stability of Phage Microgels

**Figure 5:**
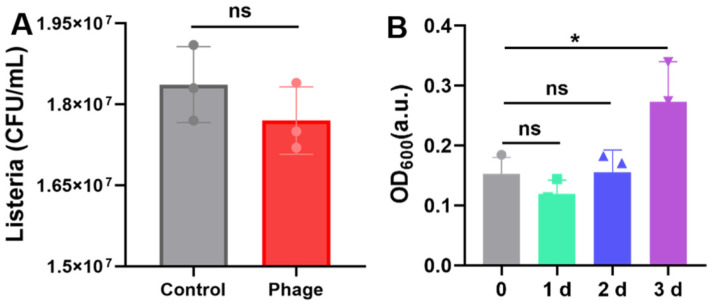
Assessment of the specificity and storage stability of phage microgels. (**A**) Anti-*Listeria* activity of the *S. typhimurium* 14,028-embedded phage microgels. (**B**) Anti-*S. typhimurium* 14,028 activity of the *S. typhimurium* 14,028-embedded phage microgels after being stored for 1, 2, and 3 days at 4 °C. *n* = 3; data are mean ± s.d. Significance was calculated by one-way ANOVA with Tukey multiple comparisons test; ns, not significant, * *p* < 0.05.

Moreover, to demonstrate the specific bactericidal activity of our microgels, we incubated phage microgels with *Listeria* at the initial titer of 10^6^ CFU mL^−1^. After culturing for 12 h, the *Listeria* concentration of the solution containing *S. typhimurium* 14,028-embedded phage microgels reached 1.77 × 10^7^ CFU/mL, which showed no significance with that of the control solution (1.84 × 10^7^ CFU/mL) (Figure 5A). The same strong growing trend with the control regardless of the participation of microgels illustrated the specific targeting of these microgels. We further investigate the phage microgels stability by storing them at different temperatures and different storage periods. As shown in Figure S4, the additions of phage microgels stored at different temperatures have largely reduced the OD_600_ intensities of *S. typhimurium* 14,028 solutions compared with the controls. The killed rates of 4 °C-stored phage microgels reached 78.7 ± 2.7%, which was higher than those stored at 25 °C (56.3 ± 3.2%), 37 °C (57.3 ± 1.7%), and 50 °C (53.4 ± 5.3%). The high anti-bacterial activity of 4 °C-stored phage microgels may be due to the high activity of proteins on the surface of phages. Moreover, the anti-bacterial activity has no obvious decrease after being stored for 2 days at 4 °C (0.155 ± 0.030) compared with that of the 0 day (0.152 ± 0.023) (Figure 5B). In summary, the optimal storage temperature should be 4 °C, and their anti-bacterial activity can be maintained for 2 days.

### 2.6. Anti-Salmonella Efficacy of Phage Microgel Spray on Chicken Meat

**Figure 6:**
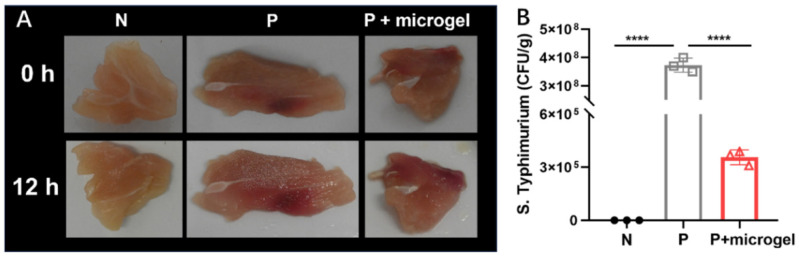
Anti-microbial activity of phage microgels on chicken samples. (**A**) Pictures of chicken samples treated with water (negative control, N), *S. typhimurium* 14,028 (positive control, P), and *S. typhimurium* 14,028 with phage microgels, (P + microgel), at 0 and 12 h. (**B**) Bacterial titer count of the collected bacterial suspension from contaminated samples (*n* = 3 independent chicken samples per group). Significance was calculated by one-way ANOVA with Tukey multiple comparisons test; **** *p* < 0.0001.

We further used these phage microgels to inhibit bacterial contamination in chicken samples. The chicken samples were first contaminated with *S. typhimurium* 14,028 at 10^7^ CFU/g, followed by spraying with phage microgels. Samples contaminated with sterile water were used as a negative control group (N). These samples were then covered with food wrap and placed in sealed petri dishes at room temperature for 12 h. Further, samples were immersed in PBS and vortexed for 2 min to collect the live bacteria. After culturing for 12 h, some microdroplets were generated on the wrap of the positive group, showing visual differentiation from other groups (Figure 6A). After culturing 12 h, the contaminated chicken samples with no microgel treatment reached an average bacteria load of 3.7 × 10^8^ CFU/g, which was significantly higher than that of the samples treated with microgels (3.6 × 10^5^ CFU/g, up to 3 log reduction). The phage-embedded microgels have killed 99.90% of the contaminated *S. typhimurium* 14,028 on chicken samples, showing a high anti-bacterial activity for food samples (Figure 6B).

## 3. Methods

### 3.1. Phage Propagation, Purification, and Concentration

(1) Phage enrichment

Phages were enriched from sewage samples. Sewage samples were collected from a local market in Tianjin, China. Each sample was centrifugated at 5000× *g* for 20 min at 4 °C to remove solid debris. To ensure sterility, the supernatants were filtered through 0.20 mm syringe filters. To enrich potential phages, 10 mL of each treated sample, 10 mL of 2 × LB broth, and 1 mL of an overnight culture of *Salmonella typhimurium* ATCC 14,028 (Tianjin University laboratory free donation) were mixed and incubated at 37 °C with shaking at 160 rpm for 16–20 h to promote phage growth. Following incubation, the medium underwent re-treatment via centrifugation and filtration for further use.

(2) Phage isolation and purification

The presence of phages in the samples was confirmed using the double-layer agar method [21], which revealed clear plaques on the host strain. Briefly, 100 mL of the enriched sample was overnight cultured with 100 mL of host strains in 3 mL of 0.7% (*w*/*v*) top LB agar (supplemented with 1 mM CaCl_2_ and 1 mM MgCl_2_), which was pre-warmed to 55 °C. This mixture was then poured onto 1.5% (*w*/*v*) LB agar plates and incubated for 16–20 h at 37 °C. Further, a distinct plaque was transferred and resuspended in 1 mL of Salt Magnesium (SM) buffer to culture for 24 h to allow for phage replication. The morphology of the purified phages was observed using the double-layer method. Purification involved at least three passages to ensure that plaques on the plate exhibited consistent size and shape.

(3) Phage propagation

For initial enrichment, the purified phages were prepared using the plate lysate method [22]. The mixture of 10^4^ PFU of purified phages and 100 mL of the overnight-cultured host strain was cultured in 3 mL of 0.7% (*w*/*v*) top LB agar (supplemented with 1 mM CaCl_2_ and 1 mM MgCl_2_) at 55 °C. This mixture was poured onto 1.5% (*w*/*v*) LB agar plates and incubated at 37 °C for 16–20 h. Subsequently, 5 mL of SM buffer was added to each plate at room temperature for 1–2 h for confluent lysis. The top agar layer was then chopped using cell scrapers, and the liquid was collected. This mixture was centrifuged at 1000× *g* for 15 min at 4 °C. The supernatant was filtered through 0.2 mm filters and titered using the spot assay method, yielding high-titer (~10^9^ PFU/mL) phage stocks.

(4) Liquid amplification

For further amplification, 1 mL of the overnight-cultured host strain was added to 50 mL of LB liquid broth (supplemented with 1 mM CaCl_2_ and 1 mM MgCl_2_) and incubated at 37 °C with shaking at 160 rpm. Once the optical density at 600 nm (OD_600_) reached approximately 0.3, purified phages were added at a multiplicity of infection (MOI) of ~0.1 and incubated at 37 °C with shaking at 160 rpm for 3 h. The culture was then treated with 5% (*v*/*v*) chloroform at room temperature for 15 min to inactivate bacteria. The aqueous phase was centrifuged at 5000× *g* for 20 min at 4 °C. The supernatant was then centrifuged at 12,000× *g* for 2 h at 4 °C, and the resulting precipitate was resuspended in 5 mL of SM buffer for 24 h and subsequently filtered through 0.20 mm syringe filters. The titer of the phage lysate was determined using the double-layer agar method [23].

### 3.2. Preparing Polystyrene Honeycomb Film

An amount of 100 mL polystyrene (5%, dissolved in chloroform solution) was evenly distributed onto a pristine glass slide within the humidity chamber to dry and cure for 20 min. The slide was removed from the chamber once the process was completed. After an additional hour, the honeycomb membrane was effortlessly peeled off for storage at room temperature.

### 3.3. Phage Microgel Preparation

First, to enhance hydrophilicity, the honeycomb membrane was treated with plasma for 5 min. Then, 50 mL of the bacteriophage–crosslinker solution was added onto the surface of the plasma-treated honeycomb membrane, which was placed on a glass slide in a petri dish. Moreover, a petri dish with water was placed in the vacuum-drying oven to create a humid environment. To thoroughly fill the template micropores with a mixture solution, the honeycomb membrane was placed in an oven and a low-pressure environment was maintained for 20 min in the dark. During this process, the mixture solution gradually gelled. Using a glass slide, the hydrogel layer was gently scraped off from the membrane’s surface, leaving a flexible composite encapsulated with microgel within the template micropores.

### 3.4. Separation of Microgels from the Honeycomb Membrane

First, transparent tape was carefully applied to the surface of the membrane and then torn off quickly. As the honeycomb membrane fractured in layers, the microgels were exposed from the spherical pores, which were located in the bottom half. The top layer of the honeycomb membrane was visible on the tape. Microgels were separated from the membranes by gently vortexing them for five minutes in sterile water. The bacteriophage microgel suspension was centrifuged at 12,000× *g* rpm at 4 °C for 10 min. After centrifugation, the supernatant was discarded, and the microgel was precipitated with 1 mL of sterile water. The resulting suspension could be directly used for further anti-microbial performance testing or as a material for bacteriophage microgel spray.

### 3.5. Microgel Preparation Efficiency

(1) Template hole and microgel size measurement

The template holes and microgels were imaged using an inverted microscope (Tecan, Spark). ImageJ software V1.8.0.112 was applied for precise measurements of the honeycomb membrane’s pore and microgel size. The diameter of the ball hole was selected to determine the diameter of the template hole. Three different regions were randomly chosen for each sample, with three images captured per region, resulting in a total of nine images. All visible holes of the obtained images were measured to gather detailed aperture data.

(2) Microgel preparation efficiency

Honeycomb film pore density is defined as the number of pores per unit area of the film. After obtaining three images of the honeycomb film using an inverted microscope, the pore density was calculated by manually counting all the pores in each image and measuring the corresponding area of the images.

The microgels obtained from the template separation were collected in 1 mL of sterile water. To calculate the number of microgels in the 1 mL suspension, we cast a 5 mL sample on a microscope slide and captured an image of the droplet that covered the entire area using the inverted microscope. Subsequently, we manually counted the number of microgels in that droplet and calculated the encapsulation efficiency of the microgels using Equation (1):

η = N_microgel_ × 200/S_template_ (1)

where η is the microgel preparation efficiency, N_microgel_ is the number of microgels in that droplet, and S_template_ is the size of the honeycomb film.

### 3.6. Anti-Microbial Test of Phage Microgel Patches on the Bacterial Lawn

In this experiment, we used the composite film as a flexible anti-bacterial patch to simulate an ordered arrangement of bacteriophage microgel monolayers. To assess the anti-bacterial effect of the bacteriophage microgels, we added 100 mL of bacterial suspension to 3 mL of LB semi-solid agar medium preheated to 50 °C and mixed them quickly and evenly. We then swiftly poured the mixture onto pre-prepared LB solid agar plates and let them sit under sterile conditions for 20 min to solidify. Subsequently, we gently placed the cleaned composite patches on the plates. Lastly, these double-layer plates were incubated at 37 °C for 18 h to observe the formation of bacteriophage plaques.

### 3.7. Anti-Microbial Test of Phage Microgel Sprays on the Bacterial Lawn

The 100 mL bacteriophage microgel suspension was evenly mixed with 100 mL bacterial suspension, added into 3 mL LB semi-solid agar medium at 50 °C, remixed evenly, quickly poured into the prepared LB solid agar plate, and left for 20 min in a sterile environment to wait for solidification. Then, the double agar plate was incubated at 37 °C for 18 h to observe the plaque status.

### 3.8. Food Decontamination Test of Phage Microgels

The egg samples were randomly cut into nine squares from the shells. The chicken samples were randomly cut into nine pieces and evenly distributed into three groups. The samples were divided into three distinct groups, labeled A, B, and C. Groups A and B were designated as control groups, while C was the experimental group. Groups B and C were contaminated with *Salmonella* suspensions to simulate a real-world scenario. The surfaces of Group A and B samples were sprayed with 200 mL of sterile water. A freshly prepared phage microgel suspension of 200 mL was uniformly sprayed onto the samples of Group C. To prevent cross-contamination, the treated samples were wrapped in plastic. The samples were incubated for a period of 12 h at room temperature to observe bacterial growth. Furthermore, the growth of bacteria was extracted from the sample surfaces by transferring the treated samples into 10 mL of sterile PBS and vortexed for 2 min. The concentration of bacteria in the samples was determined to further assess the effectiveness of the phage microgel treatment in reducing Salmonella contamination.

## 4. Conclusions

In summary, we have developed a biomolecular-friendly high-throughput method for the preparation of anti-*S. typhimurium* 14,028 phage microgels for the biocontrol of *Salmonella* contamination on food samples. This method can produce over 210,000 phage microgels in every square centimeter template, with each microgel containing 1.04 × 10^7^ phages. This simple, heat-free, and solvent-free method can maintain the biofunctions of biomolecules and proteinaceous materials and maintain a strong anti-bacterial efficiency of phages. The phage microgels could kill 99.90% of the contaminated *S. typhimurium* 14,028 on chicken samples and showed high anti-bacterial activity for food samples. This method can also be used for the preparation of various types of phage microgels for multidrug-resistant bacteria biocontrol. Further applications of phage microgels or patches in grocery store produce sections, or in household decontamination products, could effectively inhibit bacterial contamination in a human-friendly manner.

## Data Availability

Data are contained within the article.

## Supplementary Materials

The following supporting information can be downloaded at: https://www.mdpi.com/article/10.3390/ijms252211911/s1.
