# Supplementary material for: A Honeycomb Film Template-Based Method for High-Throughput Preparation of Anti-Salmonella typhimurium 14,028 Phage Microgels
Source: Int J Mol Sci. 2024 Nov 6;25(22):11911. doi: 10.3390/ijms252211911 (PMC11594076; doi:10.3390/ijms252211911)
Supplement: Supplementary file 1 [file ijms-25-11911-s001.zip › ijms-3283512-supplementary.pdf]

Supporting Information

A honeycomb film template-based method for high-throughput preparation of anti-*Salmonella typhimurium* 14028 phage microgels

Jing Wu[1,#], Tingtao An[2,#], Yaxiong Song[1], Shuo Wang[1,*]

[1] School of Medicine, Nankai University, Tianjin 300071, China;

wujing2020@nankai.edu.cn (J.W.); ysong90@nankai.edu.cn (Y.S.);

wangshuo@nankai.edu.cn (S.W.)

[2] College of Food Science and Engineering, Tianjin University of Science and Technology, Tianjin 300071, China; tingtaoan@163.com

[#] These authors (Jing Wu and Tingtao An) contribute equally to this work.

*Correspondence: wangshuo@nankai.edu.cn; Tel.: +86-22-8535-8445

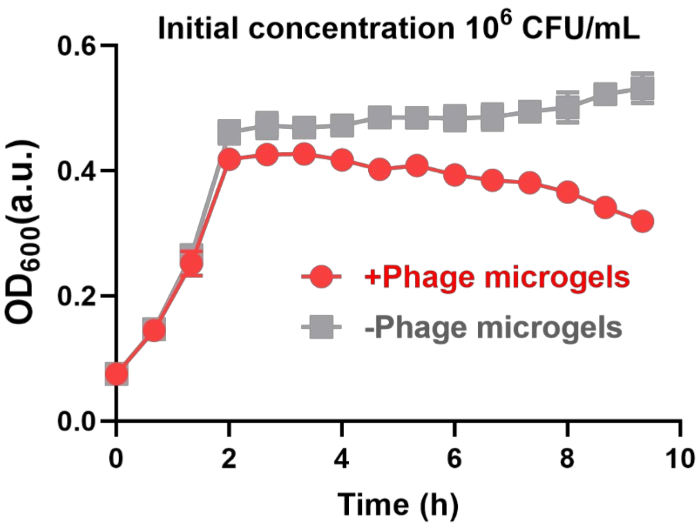

Figure S1. Comparison of kill curves of phage microgels and blank group for *S. typhimurium* 14028 suspension, incubated in TSB for 9 h (n=3).

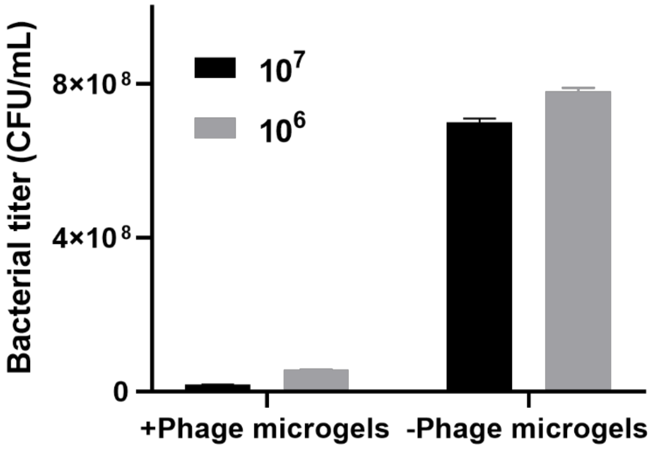

Figure S2. Final titer count of *S. typhimurium* 14028 incubated with phage microgels and without phage microgels in TSB after 9h at the initial concentrations of 10^7 and 10^6 CFU/mL (n=3).

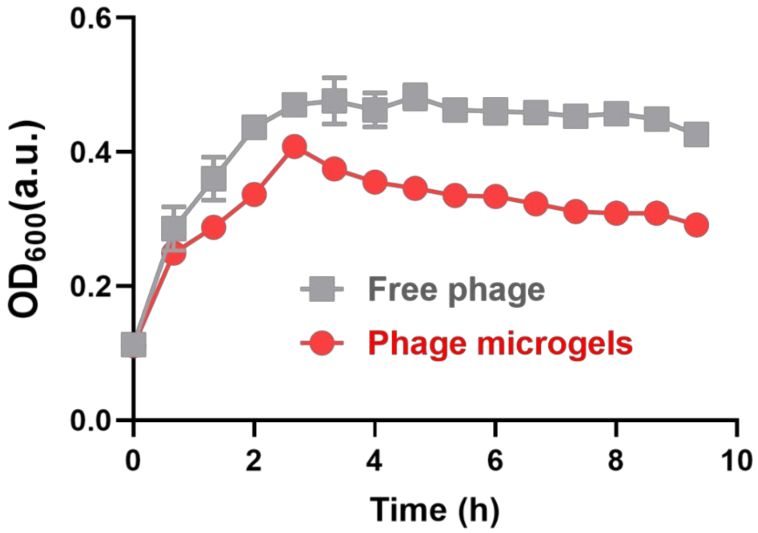

Figure S3. Kill curves for *S. typhimurium* 14028 suspension, incubated in TSB for 9h with phage and phage microgels (n=3).

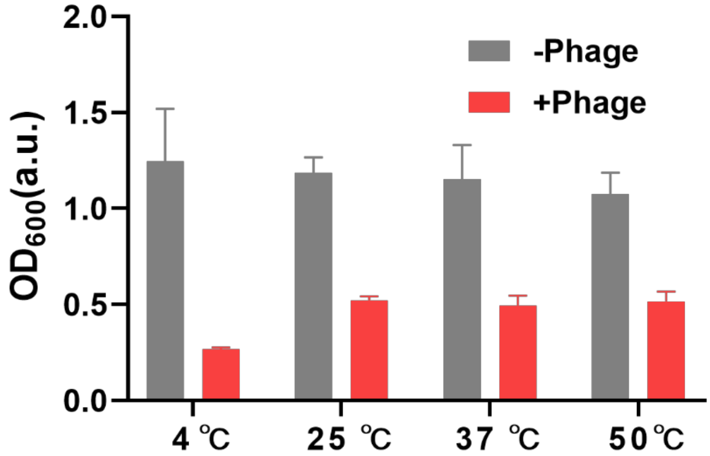

Figure S4. The OD_{600} intensities of *S. typhimurium* 14028 suspensions incubated with phage microgels at different temperatures (n=3).
